# The Adenylate Cyclase Toxins of *Bacillus anthracis* and *Bordetella pertussis* Promote Th2 Cell Development by Shaping T Cell Antigen Receptor Signaling

**DOI:** 10.1371/journal.ppat.1000325

**Published:** 2009-03-06

**Authors:** Silvia Rossi Paccani, Marisa Benagiano, Nagaja Capitani, Irene Zornetta, Daniel Ladant, Cesare Montecucco, Mario M. D'Elios, Cosima T. Baldari

**Affiliations:** 1 Department of Evolutionary Biology, University of Siena, Siena, Italy; 2 Department of Internal Medicine and Immunoallergology, University of Florence, Florence, Italy; 3 Department of Biomedical Sciences, University of Padua, Padua, Italy; 4 Unité de Biochimie des Interactions Macromoléculaires, CNRS URA 2185, Institut Pasteur, Paris, France; Johns Hopkins School of Medicine, United States of America

## Abstract

The adjuvanticity of bacterial adenylate cyclase toxins has been ascribed to their capacity, largely mediated by cAMP, to modulate APC activation, resulting in the expression of Th2–driving cytokines. On the other hand, cAMP has been demonstrated to induce a Th2 bias when present during T cell priming, suggesting that bacterial cAMP elevating toxins may directly affect the Th1/Th2 balance. Here we have investigated the effects on human CD4^+^ T cell differentiation of two adenylate cyclase toxins, *Bacillus anthracis* edema toxin (ET) and *Bordetella pertussis* CyaA, which differ in structure, mode of cell entry, and subcellular localization. We show that low concentrations of ET and CyaA, but not of their genetically detoxified adenylate cyclase defective counterparts, potently promote Th2 cell differentiation by inducing expression of the master Th2 transcription factors, c-maf and GATA-3. We also present evidence that the Th2–polarizing concentrations of ET and CyaA selectively inhibit TCR–dependent activation of Akt1, which is required for Th1 cell differentiation, while enhancing the activation of two TCR–signaling mediators, Vav1 and p38, implicated in Th2 cell differentiation. This is at variance from the immunosuppressive toxin concentrations, which interfere with the earliest step in TCR signaling, activation of the tyrosine kinase Lck, resulting in impaired CD3ζ phosphorylation and inhibition of TCR coupling to ZAP-70 and Erk activation. These results demonstrate that, notwithstanding their differences in their intracellular localization, which result in focalized cAMP production, both toxins directly affect the Th1/Th2 balance by interfering with the same steps in TCR signaling, and suggest that their adjuvanticity is likely to result from their combined effects on APC and CD4^+^ T cells. Furthermore, our results strongly support the key role of cAMP in the adjuvanticity of these toxins.

## Author Summary

Colonization by pathogens requires keeping at bay the host immune defenses, at least at the onset of infection. The adenylate cyclase (AC) toxins produced by many pathogenic bacteria assist in this crucial function by catalyzing the production of cAMP, which acts as a potent immunosuppressant. Nevertheless, at low concentrations, these toxins act as adjuvants, enhancing antibody responses to vaccination. We have investigated the molecular basis of the immunomodulatory activities of two AC toxins, *Bacillus anthracis* edema toxin and *Bordetella pertussis* CyaA. We show that high toxin concentrations inhibit activation of T lymphocytes, which orchestrate the adaptive immune response against pathogens, whereas low toxin concentrations promote differentiation of helper T lymphocytes to Th2 effectors, which are required for development of antibody-producing cells. Both the immunosuppressant and Th2–driving activities of the toxins are dependent on cAMP. The results demonstrate that, dependent on their concentration, the AC toxins of *B. anthracis* and *B. pertussis* evoke distinct responses on target T lymphocytes by differentially modulating antigen receptor signaling, resulting either in suppression of T cell activation or Th2 cell differentiation. These results are of relevance to the evolution of disease in infected individuals and provide novel mechanistic insight into the adjuvanticity of these toxins.

## Introduction

Development of an effective humoral immune response is crucially dependent on T cell help. The last step of B cell differentiation, involving immunoglobulin affinity maturation and isotype switching, occurs in peripheral lymphoid organs under the guidance of a specialized CD4^+^ T cell subset, known as T helper 2 (Th2). These cells provide both soluble (IL-4) and membrane-bound (CD40L) factors essential for terminal differentiation of antigen specific B cells [1]. Th2 cells are characterized by expression of a unique complement of cytokines, including IL-4, IL-5, IL-10 and IL-13, which are expressed through a complex transcriptional program involving chromatin remodelling at the Th2 cytokine locus control region and *de novo* expression of the lineage specific transcription factors c-maf and GATA-3 [2].

Priming the Th2 differentiation program in naive CD4^+^ T cells requires essential cues which are provided by antigen presenting cells (APC) in the form of cytokines. Engagement of the T cell antigen receptor (TCR) on naive T cells in the presence of IL-4 promotes their differentiation to Th2 effector cells, whilst simultaneously antagonising committment to the alternative Th1 lineage, which controls cell mediated immunity [1],[2]. Additional factors present during T cell priming may profoundly affect the developmental program of helper T cells. Among these, of paramount importance is the second messenger cAMP, which is produced by cellular adenylate cyclases in response to heterotrimeric G-protein coupled surface receptors, such as the receptors for prostaglandin E_2_, a proinflammatory prostanoid produced by activated APC [3]. cAMP has been shown to favour Th2 cell differentiation and GATA-3 dependent production of IL-4 and IL-5 through a pathway regulated by phosphoinositide-dependent kinase 1 (PDK1) and protein kinase A (PKA) [4]–[9].

Suppression of both innate and adaptive immune responses through elevation of intracellular cAMP to supraphysiological levels represents a powerful strategy of immune evasion by many bacterial pathogens. This can be achieved indirectly, as for the bacterial enterotoxins, cholera toxin (CT) and *E. coli* heat-labile enterotoxin (LT), which enhance intracellular cAMP production by activating the Gsα subunit of heterotrimeric G-proteins coupled to cellular adenylate cyclases [10]. Alternatively, bacteria such as *B. anthracis* or *B. pertussis* produce and deliver into target cells an adenylate cyclase toxin, the edema factor (EF) and CyaA respectively, respectively, which are themselves adenylate cyclases that catalyze the production of large amounts of cAMP [11],[12]. Notwithstanding their immmunosuppressive activity, when administered to mice at subtoxic concentrations together with antigen these toxins potentiate antibody responses, an effect associated with enhanced generation of antigen specific Th2 cells [13]–[17]. The adjuvanticity of cAMP elevating toxins is believed to result from their capacity to modulate APC differentiation and function. This is exemplified by ET and CyaA, which have been reported to selectively inhibit the production by macrophages and dendritic cells of the master Th1 polarizing cytokine, IL-12, while upregulating IL-4 and IL-10 production, thereby enhancing the induction of Th2 cells [13]–[15], [17]–[19]. The finding that both non-hydrolysable cAMP analogues and PGE_2_ evoke similar effects on APC [18]–[21] strongly supports the notion that the cAMP elevating activity of these toxins largely accounts for their capacity to differentially affect cytokine production by APC.

We and others have demonstrated that ET and CyaA potently suppress T cell activation [22]–[24]. This activity results from their capacity to uncouple TCR engagement from activation of the MAP kinase cascade, which is essential for the initiation of the transcriptional program governing T cell activation, proliferation and subsequent differentiation to armed effector cells. Here we have investigated the additional possibility, suggested by the instructive role of the cAMP/PKA axis in Th2 cell differentiation [4]–[9], that these bacterial toxins might alter TCR signaling to promote naive T cell committment to the Th2 lineage when used at low concentrations. The results show that both ET and CyaA, but not their enzymatically deficient counterparts, directly affect the Th1/Th2 balance by selectively inhibiting TCR dependent activation of the Th1 driving kinase Akt1 while enhancing activation of two essential components of the TCR signaling cascade selectively implicated in human Th2 cell differentiation, the guanine nucleotide exchanger Vav1 and the stress kinase p38. These data support the notion that the adjuvanticity of these cAMP elevating toxins results from their combined effects on APC and CD4^+^ T cells.

## Results

### Low concentrations of ET and CyaA catalyze sustained production of cAMP and induce PKA activation in T cells

**Figure 1:**
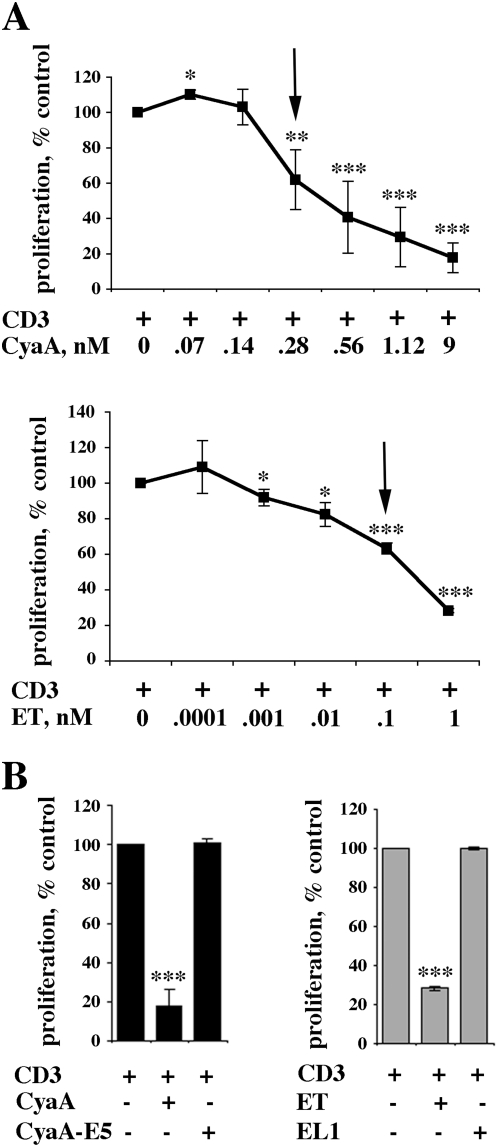
Concentration dependent suppression of T cell proliferation by CyaA and ET. (A) [^3^H]-thymidine uptake by PBL stimulated for 48 h by CD3 cross-linking in the presence or absence of the indicated concentrations of CyaA (*top*) or ET (*bottom*). The results, obtained on triplicate samples of PBL from 5 independent donors, are expressed as % [^3^H]-thymidine uptake (cpm) by CyaA or ET treated cells compared to control cells stimulated in the absence of either toxin (taken as 100%). The arrow shows the toxin concentration selected for the polarization experiments (CyaA, 0.28 nM; ET, 0.11 nM). (B) [^3^H]-thymidine uptake by PBL stimulated for 48 h by CD3 cross-linking in the presence or absence of either CyaA and ET or the respective adenylase deficient mutants (45 nM CyaA/CyaA-E5, 110 nM ET/EL1). The results are expressed as in A. ***P≤0.001; **P≤0.01; *P≤0.05. Error bars, SD.

Both *B. anthracis* ET and *B. pertussis* CyaA potently suppress T cell activation through their cAMP elevating activity [22]–[24]. To assess the potential effect of these toxins on CD4^+^ T cell differentiation, a permissive concentration of ET or CyaA was identified in a T cell proliferation assay. Genetically inactivated mutants of ET and CyaA, EL1 [25] and CyaA-E5 [26] respectively, were included as controls. Both ET and CyaA inhibited T cell proliferation in a concentration-dependent manner (Figure 1A). Conversely, neither EL1 or CyaA-E5 affected T cell proliferation (Figure 1B). The toxin concentration selected for the polarization experiments, 0.11 nM and 0.28 nM for ET and CyaA respectively, resulted in ∼40% inhibition in the proliferation assays (see arrow in Figure 1A).

**Figure 2:**
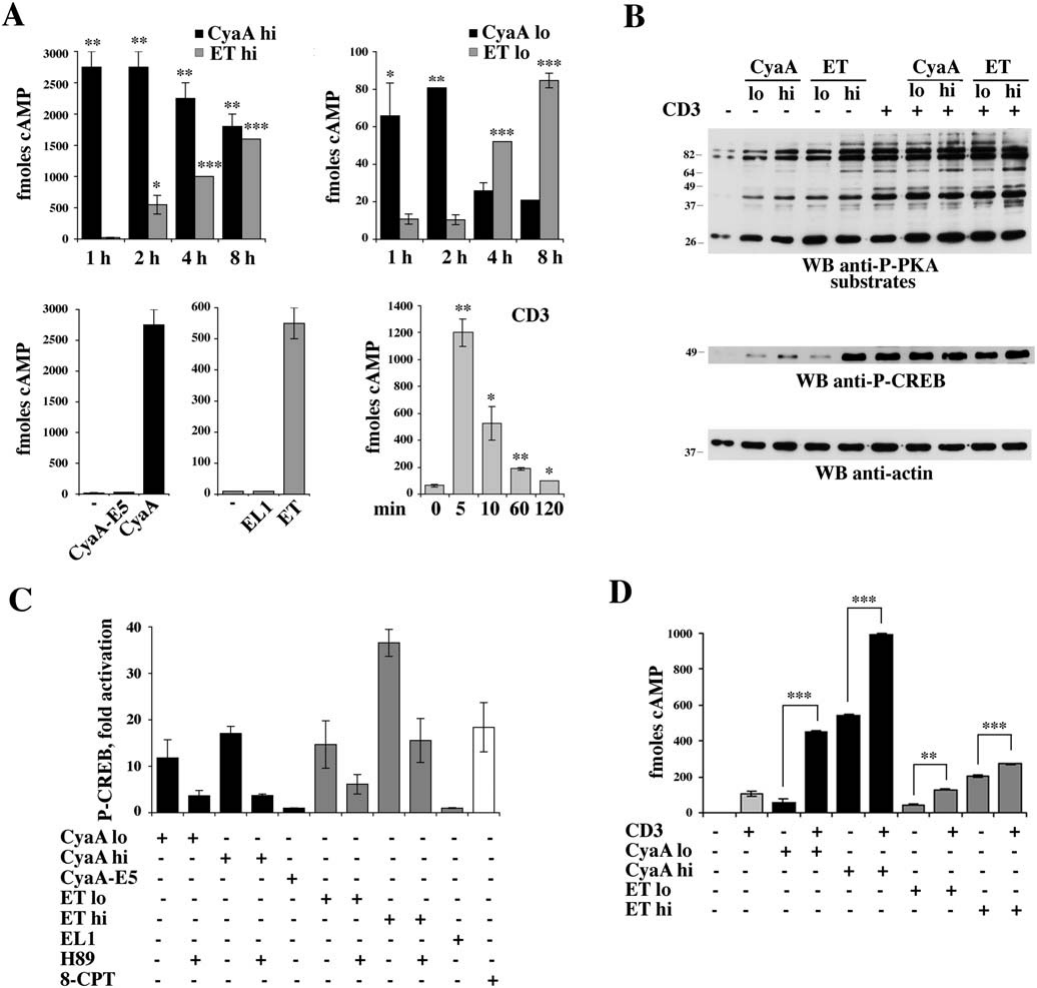
cAMP production and PKA activation in T cells treated with high and low concentrations of CyaA and ET. (A) Time course analysis of cAMP production in purified peripheral blood T lymphocytes treated with high (CyaA hi, 45 nM ; ET hi, 110 nM) (*top left*) or low (CyaA lo, 0.28 nM; ET lo, 0.11 nM) (*top right*) concentrations of CyaA or ET, or activated by TCR/CD3 cross-linking (*bottom right*). The histogram on the bottom left panel also includes the quantification of cAMP in lysates of T cells treated with the adenylase cyclase deficient CyaA and ET mutants (45 nM CyaA-E5, 110 nM EL1) for 2 h or 6 h, respectively. The results, which show the levels of cAMP measured in T cell lysates, are expressed as fmoles/10^6^ cells. Representative experiments, each carried out on duplicate samples from individual healthy donors, are shown (*n*≥4). (B) *Top*, Immunoblot analysis of the phosphorylation state of PKA substrates in post-nuclear supernatants of T cells treated with 45 nM CyaA (CyaA hi) or 0.28 nM CyaA (CyaA lo), or 110 nM ET (ET hi) or 0.11 nM ET (ET lo), for 2 h (CyaA) or 6 h (ET), and then lysed as such or after stimulation for 1 min with anti-CD3 mAb (CD3). A sample stimulated with anti-CD3 mAb alone was also included. The immunoblot was carried out using an antibody which recognizes a phosphorylated PKA consensus sequence (see Materials and Methods). The stripped filter was reprobed with a phosphospecific antibody which recognizes the active form of CREB *(middle)*. The fold activation of CREB in CyaA/ET treated samples *vs* untreated control in the experiment shown was the following: CyaA low, 8.3; CyaA high, 19.0; ET low, 8.7; ET high, 79.9. The levels of phospho-CREB in the samples treated with CyaA or ET in combination with anti-CD3 mAb *vs* samples treated with anti-CD3 mAb alone (taken as 100%) were the following: CyaA low+CD3, 98.1±4.8%; CyaA high+CD3, 103.4±8.7%; ET low+CD3, 102.1±3.2%; ET high+CD3, 112.1±8.1% (*n* = 3). A control anti-actin blot is shown below. None of the treatments modified the expression levels of CREB (data not shown). Representative experiments are presented (*n*≥3). The migration of molecular mass markers is indicated. (C) Quantification of CREB phosphorylation in post-nuclear supernatants of T cells treated with 45 nM CyaA (CyaA hi)/CyaA-E5 or 0.28 nM CyaA (CyaA lo), or 110 nM ET (ET hi) /EL1 or 0.11 nM ET (ET lo), for 2 h (CyaA) or 6 h (ET). Where indicated, cells were pretreated for 1 h with 20 µM H89. A sample stimulated for 30 min with 100 µM 8-CPT was included as positive control. The data were obtained by laser densitometry of anti-phospho-CREB immunoblots. The results are expressed as relative CREB phosphorylation (fold activation *vs* untreated controls) (*n* = 2). (D) Quantification of cAMP in lysates of T cells treated as in B. The results, which show the levels of cAMP measured in T cell lysates, are expressed as fmoles/10^6^ cells. A representative experiment, carried out on duplicate samples from an individual healthy donor, is shown (*n* = 3). ***P≤0.001; **P≤0.01; *P≤0.05. Error bars, SD.

A time course analysis of intracellular cAMP production in purified peripheral blood T cells showed that these concentrations of ET and CyaA induced a detectable accumulation of intracellular cAMP, albeit at much lower levels as compared to that obtained with high toxin concentrations (Figure 2A, top). The kinetics of cAMP production by ET and CyaA were significantly different. A fully immunosuppressive ET concentration resulted in a slow increase in intracellular cAMP beginning from 2 h, with a further progressive rise up to 8 h (Figure 2A, top left). On the other hand, a fully immunosuppressive concentration of CyaA evoked a rapid rise of cAMP to plateau levels beginning from the earliest time point analyzed, and the levels of cAMP remained high up to 8 h (Figure 2A, top left). High cAMP concentrations were still measurable after 24 h (data not shown). The kinetics of cAMP production by immunosuppressive concentrations of ET and CyaA were largely reproduced by the low toxin concentrations selected for the studies on T cell polarization (Figure 2A, top right, and data not shown for 24 h). No increase in cAMP was elicited by EL1 and CyaA-E5, even at the highest concentration used (Figure 2A, bottom left).

To understand whether the modest increase in the levels of cAMP catalyzed by low concentrations of ET or CyaA was sufficient to elicit a biological response, we measured the activity of PKA, one of the major cellular targets of cAMP. As a readout of PKA activation we used an antibody specific for the phosphorylated PKA consensus, R-X-X-pT-X-X/R-R-X-pS-X-X, which recognizes phosphorylated PKA substrates. The increase in intracellular cAMP following T cell treatment with high concentrations of ET or CyaA resulted in a strong potentiation of PKA activity, as shown by the qualitative and quantitative changes in the phosphoprotein pattern in lysates from toxin-treated cells compared to untreated cells (Figure 2B, top panel). A similar enhancement in PKA activity was also observed in cells treated with low concentrations of ET or CyaA, despite the smaller increase in intracellular cAMP measured under these conditions (Figure 2B, top panel). Interestingly, notwithstanding the different interacellular localization of the two adenylate cyclase toxins, there was a general overlap in the phosphoprotein pattern observed in cells treated with ET or CyaA. Consistent with the agonistic activity of ET and CyaA on PKA, analysis of the phosphorylation state of the transcriptional activator CREB, a specific PKA substrate, showed that low toxin concentrations induced CREB phosphorylation, albeit to a lesser extent compared to high toxin concentrations (Figure 2B, middle panel). The agonistic effect of the toxins was abrogated to a significant extent when cells were pretreated with pharmacological PKA inhibitors (H89 or KT5720) (Figure 2C and data not shown). Moroever, no CREB phosphorylation was observed in T cells treated with the adenylate cyclase defective ET or CyaA mutant (Figure 2C), supporting the notion that the effects of the toxins are mediated by the cAMP/PKA axis. Of note, maximal activation of both PKA and CREB was observed in cells stimulated by TCR/CD3 cross-linking (Figure 2B), consistent with the potent agonistic activity of the receptor on cAMP production (Figure 2A, bottom right). However, as opposed to the long-lasting increase in cAMP elicited by the toxins, TCR engagement resulted in a transient increase in intracellular cAMP (Figure 2A, bottom right). No further significant enhancement in TCR-dependent PKA or CREB activation was observed in T cells treated with ET or CyaA (Figure 2B and respective legend), despite the increase in cAMP production observed under these conditions (Figure 2D), indicating that PKA and CREB activation reaches plateau levels in response to the cAMP burst elicited by the TCR.

### ET and CyaA promote Th2 cell differentiation

**Figure 3:**
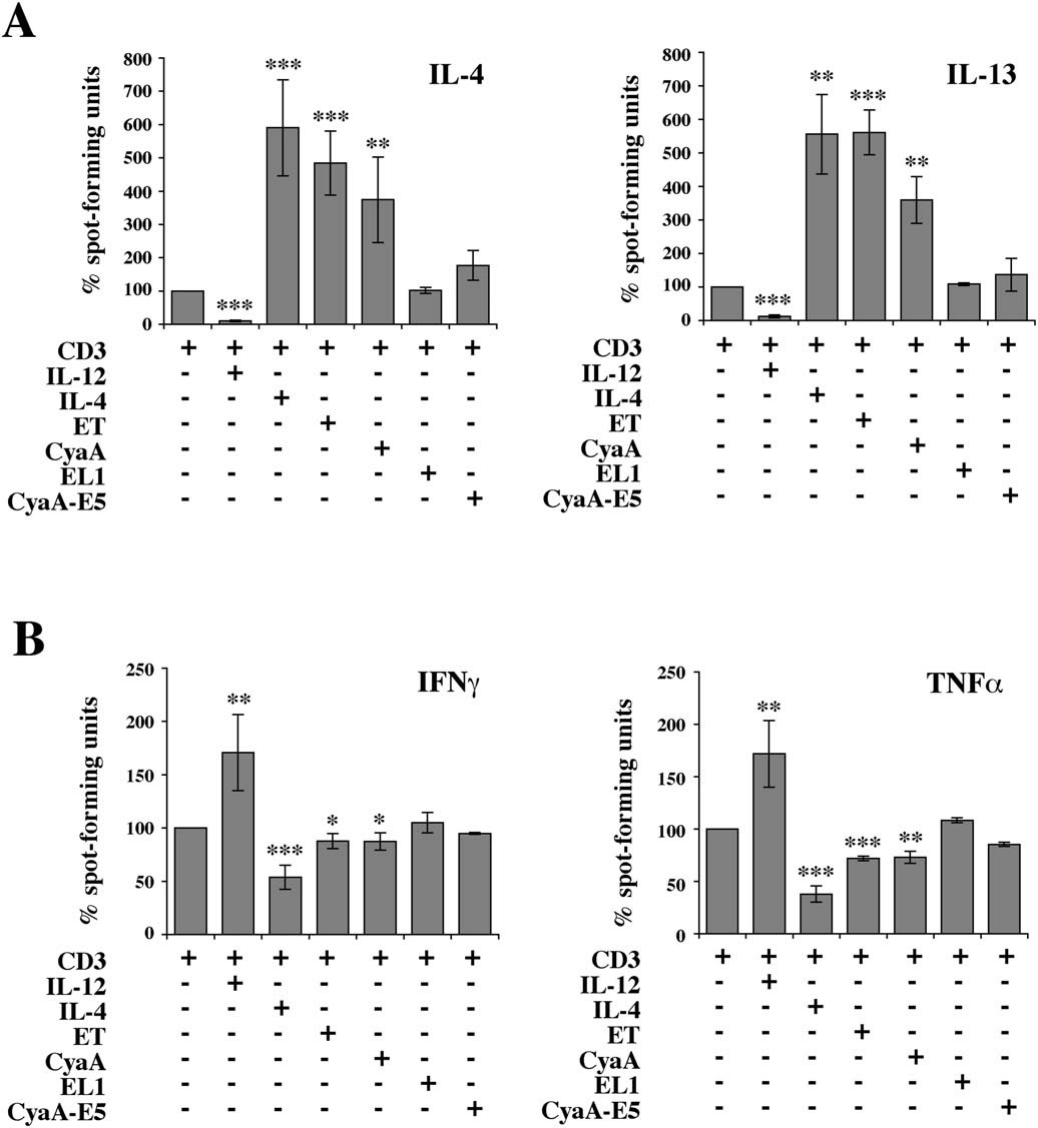
CyaA and ET promote Th2 cell differentiation through their cAMP elevating activity. Enriched CD4^+^ T cells from 6 healthy donors were primed with anti-CD3 mAb, as such or following pretreatment for 2 h with 0.28 nM CyaA/CyaA-E5, or with 0.11 nM ET/EL1. Cells primed in Th2- (IL-4) or Th1- (IL-12) inducing conditions were included as controls. After 10 days cells were washed and restimulated with anti-CD3 mAb for 48 and 24 h respectively, and the levels of IL-4 and IL-13 (A) and IFN-γ and TNF-α (B) were quantified by ELISPOT. The results, obtained on duplicate samples, are expressed as % spot-forming units by CyaA or ET treated cells compared to control cells primed in the absence of either toxin (taken as 100%). ***P≤0.0001; **P≤0.001; *P≤0.01. Error bars, SD.

To assess the impact of low concentrations of ET and CyaA on human helper T cell polarization, enriched human CD4^+^ T cells from healthy donors were exposed to ET or CyaA and subsequently primed by TCR/CD3 cross-linking using immobilized anti-CD3 mAb. After 10 days, cells were washed, and restimulated for 24 h or 48 h using the same anti-CD3 mAb. The identity of the Th subset into which cells had differentiated was determined by ELISPOT analysis of cytokine production. As shown in Figure 3A, priming of cells that had been exposed to low concentrations of ET or CyaA resulted in a dramatic increase in production of the Th2 cytokines, IL-4 and IL-13, to levels close to those measured in cells primed to differentiate to the Th2 subset (TCR/CD3 cross-linking in the presence of IL-4). Conversely, consistent with the mutual antagonism of the Th1/Th2 differentiation programs, ET or CyaA had a modest inhibitory effect on production of the Th1 cytokines IFNγ and TNF-α (Figure 3B). No significant enhancement in Th2 cytokines above the levels produced by T cells primed in neutral conditions (TCR/CD3 cross-linking alone) was observed when cells were pretreated with the adenylate cyclase defective EL1 or CyaA-E5 mutants (Figure 3A), indicating that the Th2 driving activity of ET and CyaA is dependent on their capacity to produce cAMP.

**Figure 4:**
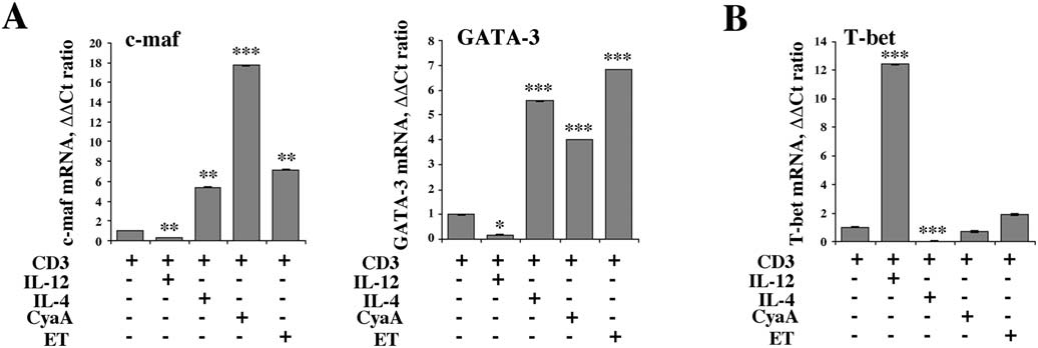
CyaA and ET promote c-maf and GATA-3 expression in primed T cells. Enriched CD4^+^ T cells from 3 healthy donors were primed with anti-CD3 mAb, as such or following pretreatment for 2 h with 0.28 nM CyaA or 0.11 nM ET. Cells primed in Th2- (IL-4) or Th1- (IL-12) inducing conditions were included as controls. After 10 days cells were restimulated with anti-CD3 mAb for 24 h. The levels of mRNA encoding c-maf and GATA-3 (A) and T-bet (B) were quantified by real-time RT–PCR. Transcript levels were normalized to the expression level of GAPDH. Syber green runs were performed with cDNAs from the same reverse transcription reaction from 400 ng of total RNA. The ΔΔC_T_ method was applied as a comparative method of quantification, using cells primed in neutral conditions (anti-CD3 mAb) as reference. The data are representative of 3 independent experiments, each in duplicate. ***P≤0.00001; **P≤0.0001; *P≤0.001. Error bars, SD.

Differentiation of helper T cells to the Th2 subset is crucially dependent on expression of the lineage specific transcription factors c-maf and GATA-3, which are essential for transcriptional regulation of the Th2 cytokine control locus [2]. To understand whether ET and CyaA promote production of IL-4 and IL-13 by shaping the transcriptional program triggered by the TCR in naive T cells, resulting in expression of lineage specific transcription factors, the levels of c-maf and GATA-3 mRNA in T cells primed in the presence of either toxin were measured by real-time RT-PCR. Both ET and CyaA potently upregulated expression of c-maf and GATA-3 to levels comparable or higher than those detectable in T cells primed in the presence of IL-4 (Figure 4A). Conversely, expression of the Th1 lineage specific transcription factor T-bet was not significantly affected in T cells primed in the presence of either CyaA or ET (Figure 4B).

### Low concentrations of ET and CyaA selectively impair TCR–dependent activation of Akt1, while potentiating activation of Vav1 and p38

**Figure 5:**
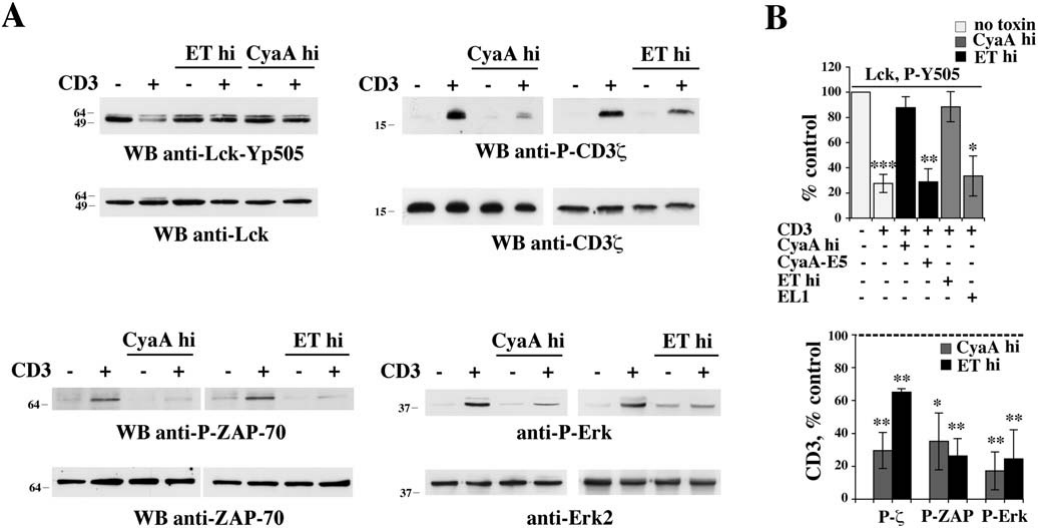
Immunosuppressive concentrations of CyaA or ET prevent initiation of TCR signaling. (A) *Top left*, Immunoblot analysis, using a phosphospecific antibody, of Lck phosphorylation on the inhibitory C-terminal tyrosine residue (Y505) in postnuclear supernatants from PBL activated for 1 min by CD3 cross-linking in the presence or absence of either 45 nM CyaA or 110 nM ET (CyaA hi, ET hi). *Top right*, Immunoblot analysis, using an anti-phosphotyrosine antibody, of CD3ζ specific immunoprecipitates from PBL treated as above. *Bottom*, Immunoblot analysis, using phosphospecific antibodies, of ZAP-70 (left) and Erk1/2 (right) phosphorylation in postnuclear supernatants from PBL activated for 1 min (ZAP-70) or 5 min (Erk1/2) by CD3 cross-linking in the presence or absence of 45 nM CyaA or 110 nM ET (CyaA hi, ET hi). (B) Quantification by laser densitometry of the relative levels of Lck (phosphorylation in unstimulated cells taken as 100%), or CD3ζ, ZAP-70 and Erk1/2 phosphorylation (phosphorylation in anti-CD3 stimulated cells taken as 100%, indicated as a dotted line) in PBL activated by CD3 cross-linking in the presence of 45 nM CyaA or 110 nM ET (CyaA hi, ET hi) (*n*≥3). Where indicated, cells were activated in the presence of the adenylase cyclase deficient CyaA and ET mutants (45 nM CyaA-E5, 110 nM EL1) for 2 h or 6 h, respectively (*n* = 2). ***P≤0.001; **P≤0.01; *P≤0.05. Error bars, SD.

**Figure 6:**
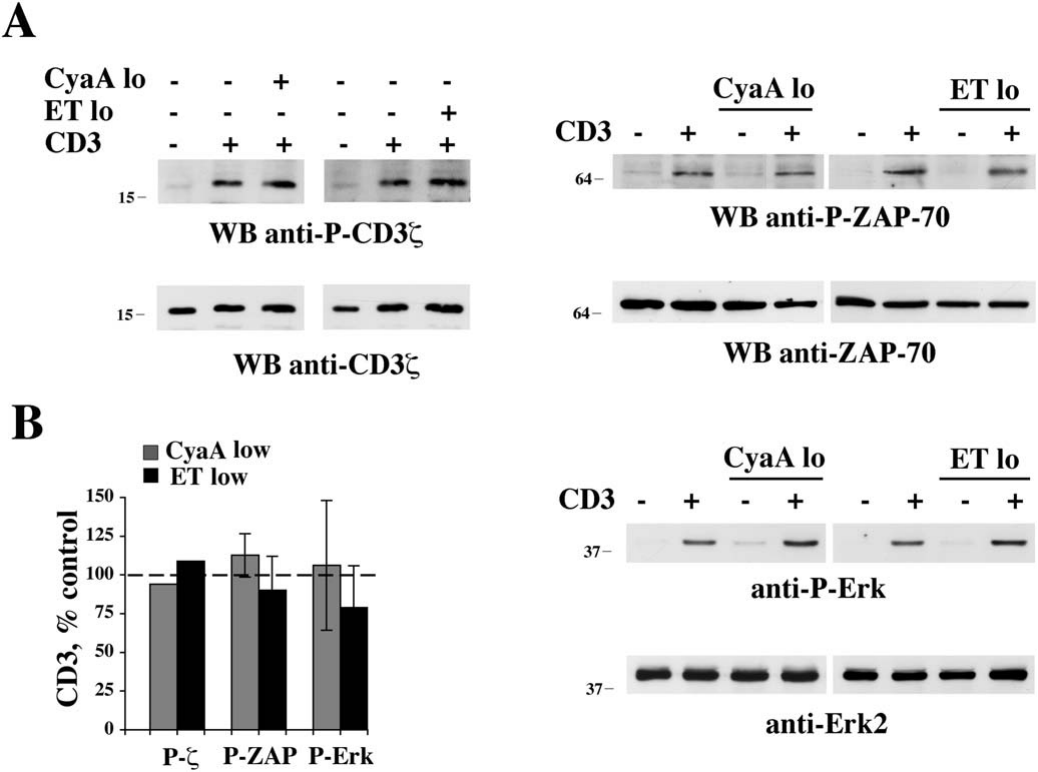
Low CyaA or ET concentrations do not affect initiation of TCR signaling. (A) Immunoblot analysis, using phosphospecific antibodies, of CD3ζ, ZAP-70, or Erk1/2 phosphorylation in postnuclear supernatants of cells activated as above in the presence or absence of either 0.28 nM CyaA or 0.11 nM ET (CyaA lo, ET lo). Filters were stripped and re-probed with control antibodies. Representative experiments are shown (*n*≥3). The migration of molecular mass markers is indicated. (B) Quantification by laser densitometry of the relative levels of CD3ζ, ZAP-70, and Erk1/2 phosphorylation (phosphorylation in anti-CD3 stimulated cells taken as 100%, indicated as a dotted line) in PBL activated by CD3 cross-linking in the presence or absence of either 0.28 nM CyaA or 0.11 nM ET (CyaA lo, ET lo) (*n*≥3). Error bars, SD.

TCR signaling is initiated by Lck, a T cell specific Src family protein tyrosine kinase which is responsible for phosphorylation of the ITAMs within the ζ chain of the CD3 complex. As all Src kinases, Lck is negatively regulated by a C-terminal tyrosine residue, Y505, which, when phosphorylated, establishes an intramolecular interaction with the SH2 domain, resulting in a close, inactive conformation. The inhibitory tyrosine residue is phosphorylated by Csk, which in resting cells is maintained close to Lck in lipid rafts through interaction with the PAG adaptor and whose activity is potentiated by PKA dependent phosphorylation of a serine residue at position 364 [27]. By elevating intracellular cAMP, ET and CyaA have therefore the potential to antagonize TCR signaling beginning from the earliest step. Analysis of the phosphorylation state of Y505 on Lck using a phosphospecific antibody revealed that high concentrations of ET or CyaA effectively block TCR dependent dephosphorylation of Lck (Figure 5A and 5B). This activity was not reproduced by the respective adenylate cyclase defective mutants (Figure 5B), supporting the notion that the suppressive effect of ET and CyaA on TCR dependent Lck activation is mediated by cAMP. No enhancement in Lck kinase activity in response to TCR engagement was moreover observed when cells were pretreated with high concentrations of ET or CyaA, as assessed by measuring Lck autophosphorylation in *in vitro* kinase assays (data not shown). Consistent with the failure of the TCR to trigger activation of Lck in the presence of either toxin, both TCR dependent CD3ζ phosphorylation and activation of the effector kinase ZAP-70, which occurs following recruitment to the phosphorylated ITAMs of CD3ζ, were found to be inhibited by ET or CyaA (Figure 5A and 5B). In agreement with previous reports [22]–[24], activation of the MAP kinase cascade, which couples these early signaling events to gene transcription, was found to be impaired by immunosuppressive concentrations of ET or CyaA, as assessed using as a readout phosphorylation of Erk1/2 (Figure 5A and 5B). Conversely, neither CD3ζ and ZAP-70 phosphorylation, nor Erk1/2 phosphorylation, were affected when the TCR was stimulated in cells exposed to low, Th2 polarizing concentrations of ET or CyaA (Figure 6A and 6B).

**Figure 7:**
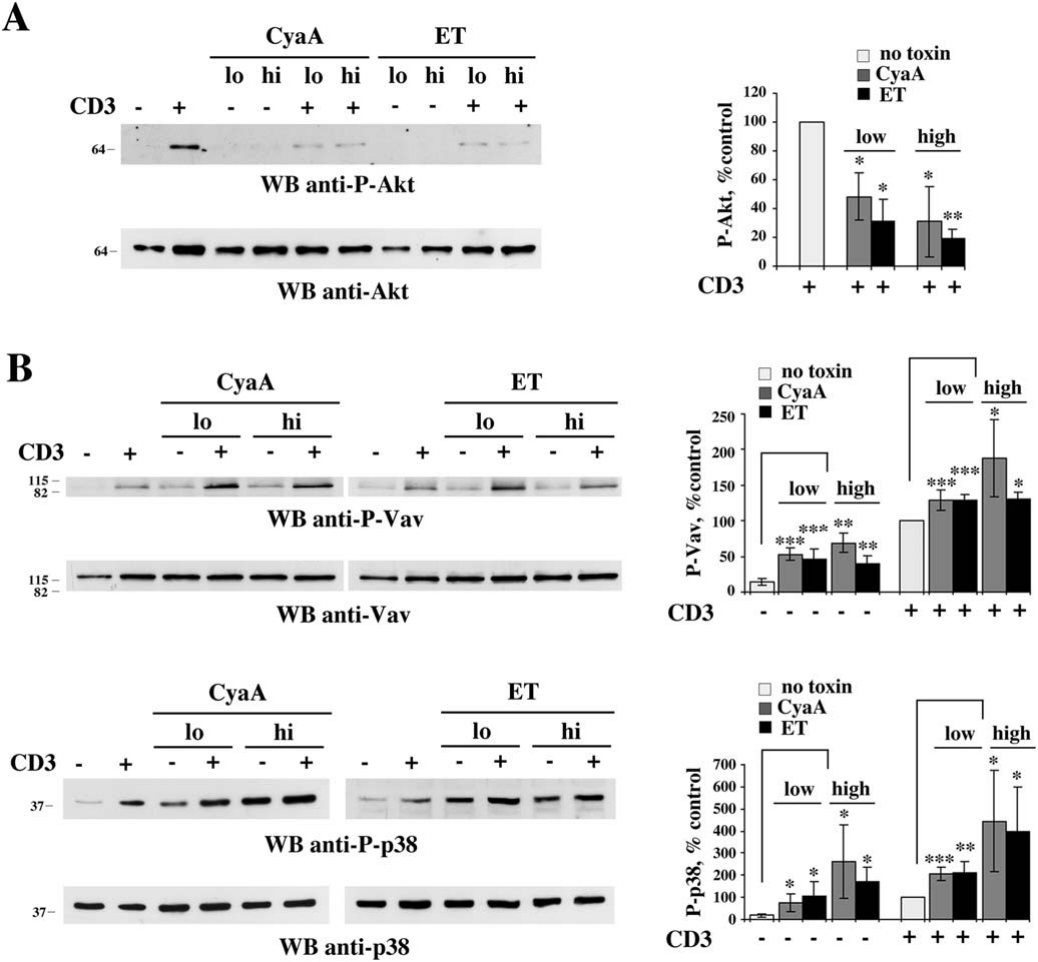
Low CyaA or ET concentrations impair TCR–dependent Akt1 phosphorylation while enhancing Vav1 and p38 phosphorylation. (A) Immunoblot analysis, using a phosphospecific antibody, of Akt1 activation in postnuclear supernatants from PBL activated for 5 min by CD3 cross-linking in the presence or absence of 45 nM CyaA or 110 nM ET (CyaA hi, ET hi) or alternatively in the presence or absence of 0.28 nM CyaA or 0.11 nM ET (CyaA lo, ET lo). A representative experiment is shown (*n* = 3). (B) Immunoblot analysis, using phosphospecific antibodies, of Vav1 (*top*) or p38 (*bottom*) activation in postnuclear supernatants from PBL activated for 1 min (Vav) or 5 min (p38) by CD3 cross-linking in the presence or absence of 45 nM CyaA or 110 nM ET (CyaA hi, ET hi) or alternatively in the presence or absence of 0.28 nM CyaA or 0.11 nM ET (CyaA lo, ET lo). Filters were stripped and re-probed with control antibodies. Representative experiments are shown (*n*≥4). The migration of molecular mass markers is indicated. The graphs on the right of the immunoblots show the quantification by laser densitometry of the relative levels of Akt1, Vav1, and p38 phosphorylation (phosphorylation in anti-CD3 stimulated cells taken as 100%) in PBL activated by CD3 cross-linking in the presence or absence of 45 nM CyaA or 110 nM ET (CyaA hi, ET hi) or alternatively in the presence or absence of 0.28 nM CyaA or 0.11 nM ET (CyaA lo, ET lo). ***P≤0.001**P≤0.01; *P≤0.05. Error bars, SD.

To understand whether Th2 polarizing concentrations of ET and CyaA could selectively affect downstream components of TCR signaling specifically implicated in Th lineage committment, we focused on two molecules in the TCR signaling cascade, the Rac/Cdc42 specific guanine nucleotide exchanger Vav1 and the stress-activated kinase p38, which have been implicated in human Th2 cell differentiation [28]–[32]. Furthermore, we assessed the effect of the toxins on the serine/threonine kinase Akt1, which has been associated to Th1 cell differentiation [9]. Strikingly, analysis of Akt1 activation using phosphospecific antibodies which recognize two critical residues, T308 and S473, showed that low, Th2 polarizing concentrations of ET or CyaA were sufficient to potently impair TCR dependent Akt phosphorylation (Figure 7A and data not shown). Conversely, both basal and TCR dependent Vav1 phosphorylation on Y174, which positively regulates Vav1 activity, was potentiated by low concentrations of ET or CyaA (Figure 7B). A similar enhancement was observed for p38 (Figure 7B), consistent with the capacity of PKA to act as an agonist of this kinase [31],[33]. The phosphodiesterase inhibitor, IBMX, further potentiated the agonistic activity of the toxins on p38 activation (data not shown), further supporting the notion that the effects of the toxins are mediated by cAMP. Hence ET and CyaA alter the Th1/Th2 balance at least in part by antagonizing the Akt1 dependent pathway leading to Th1 cell differentiation and by potentiating the Vav1 and p38 dependent pathway(s) leading to Th2 cell differentiation. Of note, TCR dependent Vav1 and p38 activation was not impaired, but actually enhanced, when cells were pretreated with high concentrations of ET or CyaA (Figure 7B), despite their potent inhibitory activity on initiation of TCR signaling, suggesting that an Lck independent pathway triggered by the TCR, which can be potentiated by cAMP, may contribute to a significant extent to their activation.

## Discussion

The *B. anthracis* ET and *B. pertussis* CyaA adenylate cyclase toxins act as potent suppressors of T cell activation and proliferation in the 10^−9^–10^−6^ molar range of concentrations [22]–[24]. In the absence of systemic intoxication, these high concentrations are likely to be reached only locally through accumulation of the toxins at the primary site of infection. However, there are anatomical districts and localized infections (*e.g.* cutaneous anthrax) where low amount of toxins may be released and might modulate the host immune response. We found that both ET and CyaA are potent promoters of naive CD4^+^ T cell differentiation to Th2 effectors when used at subnanomolar concentrations (0.1–0.3 nM). Interestingly, distinct effects of high *vs* low concentrations of CyaA have also been observed in neutrophils and other phagocytes, ranging from cytolysis to apoptosis to impairment of effector functions [34], suggesting the biological outcome of host cell exposure to the toxin is likely to be dictated by its proximity to the bacterium. The sensitivity of T cells to such low ET concentration can be accounted for by the fact that human leukocytes express the high affinity CMG2 receptor for protective antigen (PA), the receptor binding subunit of ET [35]. Furthermore, although T cells lack CD11b/CD18, the only known CyaA receptor, CyaA can effectively insert into cell membranes or artificial lipid bilayers in the absence of CD11b/CD18, albeit with a reduced efficacy [36]. The presence on T cells of a putative alternative CyaA receptor cannot however be ruled out.

The immunosuppressant activity of high concentrations of ET and CyaA is fully consistent with the known inhibitory effects of cAMP on T cell activation. In physiological conditions cAMP production by a TCR-coupled adenylate cyclase is part of a negative feed-back loop which ensures extinction of TCR signaling through PKA dependent activation of Csk, a kinase that inhibits Lck by phosphorylating its C-terminal tyrosine residue [27]. This feed-back loop does not become immediately operational because cAMP production is counterbalanced by TCR dependent recruitment of PDE-4 to lipid rafts, where also the activated TCR localizes, thereby allowing the protein tyrosine kinase cascade to start [37]. Once PDE-4 dissociates from lipid rafts, the feed-back loop can terminate the signal. Since cAMP production and PKA activation are TCR-dependent, cAMP returns to basal levels after signal extinction. Alterations in this finely regulated cAMP balance by adenylate cyclase agonists, such as PGE_2_ receptors, result in impaired TCR signaling and T cell activation [27]. The inhibitory activity of high concentrations of ET and CyaA on Lck activation and CD3ζ phosphorylation indicates that these toxins preventing firing of the TCR signaling cascade by altering the cAMP balance through the massive and sustained production of cAMP. In this context, it should be underlined that TCR engagement results in a rapid elevation in the levels of cAMP, which elicit a potent enhancement in PKA activation, comparable to the one observed in cells exposed to high toxin concentrations. Nevertheless, under these conditions the TCR triggers a productive signaling cascade, as opposed to cells pre-exposed to high concentrations of ET or CyaA, supporting the importance of the spatiotemporal regulation of cAMP in TCR signaling. Low toxin concentrations on the other hand, do not inhibit initiation of TCR signaling. The intracellular concentration of cAMP measured under these conditions, which is very modest compared to the burst of cAMP evoked by the TCR, may be locally and transiently neutralized by PDE-4.

At variance with their inability to impair initiation of TCR signaling, low concentrations of ET or CyaA were found to selectively affect specific downstream nodes -Akt1, Vav1 and p38 activation- crucial to Th1/Th2 lineage committment. This activity is likely to result from their PKA dependent modulation of intracellular signaling mediators implicated in Th2 cell differentiation downstream of signal initiation. Akt1 has been reported to favour Th1 cell differentiation by providing the CD28 costimulatory signal required for expression of the Th1 cytokines IL-2 and IFN-γ [9]. Although our study was carried out on T cells stimulated by TCR/CD3 cross-linking in the absence of CD28 costimulation, the results show that Akt1 is effectively phosphorylated in response to TCR engagement and that this event is potently inhibited by low concentrations of ET or CyaA. The negative regulation of Akt by PKA [38],[39] is likely to underlie this inhibitory, TCR-distal effect of the two toxins, which would moreover favour differentiation to the Th2 lineage by potentiating the PDK1/PKA pathway coupling the TCR to IL-4 gene transcription [9].

Under the same conditions, both toxins enhance TCR dependent phosphorylation of the guanine nucleotide exchanger Vav1 and activation of the stress kinase p38, which participate in Th2 lineage commitment. A skewing of the Th1/Th2 balance to Th1, as well as defects in Th2 dependent B cell responses, have been indeed observed in Vav1^−/−^ mice [28],[40]. Furthermore, p38 has been implicated in human Th2 cell differentiation, at least in part through its capacity to promote activation of GATA-3 [29]–[32]. The similar enhancement of TCR-dependent Vav and p38 activation in the presence of high toxin concentrations, which block signal initiation, supports a local effect on a specific signaling module independent of the TCR proximal, Lck-dependent signaling cascade. While there is evidence for an agonistic role of cAMP in p38 activation [31], the potential function of cAMP in the modulation of Vav1 activity has not been directly addressed. We have previously characterized a Fyn dependent, Lck independent pathway linking the TCR to Vav1 phosphorylation and p38 activation which could be potentiated by PGE_2_ [41]. Together with the evidence implicating Vav1 and p38 in Th2 cell differentiation, the Th1 bias observed in Fyn^−/−^ mice [42],[43] may suggest a potential involvement of this cAMP sensitive pathway in the Th2 promoting activity of ET and CyaA.

It is noteworthy that the effects of ET and CyaA are almost undistinguishable, notwithstanding their differences in stucture, mode of cell entry and intracellular localization. ET is an A–B type toxin, consisting of a cell binding component, PA, which targets cells *via* the receptors TEM8 or CMG2, and a toxin component, EF. ET enters the cell by receptor mediated internalization and is transported to the endosomes, wherefrom it is released into the cytosol [11]. CyaA is a single polypeptide which binds to target cells both directly and through a membrane receptor, which in macrophages and other APC is the integrin CD11b/CD18 [44]. Following binding, the adenylate cyclase N-terminal domain is translocated accross the plasma membrane of target cells [12]. Hence EF and CyaA produce cAMP not only with different kinetics, which is delayed for ET probably due to the multistep mechanism of delivery into host cells, but also at different subcellular locations, in the cytosol with a prevalent perinuclear localization, and close to the plasma membrane, respectively [45]–[47]. The role of AKAPs in segregation of PKA pools at specific subcellular localizations and dynamic recruitment of phosphodiesterases underscores the importance of the spatiotemporal control of cAMP signaling [48]. By focalizing cAMP production at distinct sites within the cell, EF and CyaA could differentially affect early and late events in TCR signaling. Our findings indicate that the critical targets of the cAMP dependent Th2 polarizing activities of ET and CyaA can be activated independently of the subcellular site of cAMP production. It is likely that the sustained cAMP production overrides the negative local feedback mechanisms, resulting in loss of compartmentalization of cAMP dependent signaling. It should be however underlined that low ET concentrations are almost as effective as high concentrations in triggering PKA activation, suggesting that ET may modulate other functions, such as CREB mediated gene expression [45],[47], through activation of specific PKA pools.

Both ET and CyaA have been reported to potentiate antibody responses and development of antigen specific Th2 cells in mice when coadministered with antigen [13]–[17]. This adjuvant activity had been related to their capacity to modulate cytokine production by dendritic cells and macrophages *in vitro*, resulting in reduction in IL-12 and enhancement in IL-10 and IL-4 expression [13]–[16],[18],[19]. Our finding that ET and CyaA directly affect the Th1/Th2 balance by favouring Th2 cell development suggests that their adjuvanticity is also due to their effects on CD4^+^ T cells. This activity appears mediated by cAMP, as it cannot be reproduced by the respective enzymatically deficient mutants. The Th2 polarizing effects of these toxins resulting from their modulation of cytokine expression by APC have also been related to their cAMP elevating activity [15],[18],[19],[49]. Consistent with these findings, both non-hydrolysable cAMP analogues and PGE_2_ have been reported to favour Th2 cell development both by directly affecting CD4^+^ T cell differentiation [4],[6],[8] and by shaping the pattern of cytokine production by APC [19]–[21]. The Th2 driving activity of ET may be very relevant to cutaneous anthrax, where there is a limited toxin production and where resolution of infection has been causally linked to the development of an antibody response against the toxin [50],[51].

As opposed to the clear-cut role of cAMP in both the direct and the APC dependent Th2 driving activity of ET or CyaA *in vitro*, the role of cAMP in the adjuvanticity of the toxins *in vivo* is more controvertial. While the adjuvant activity of adenylate cyclase deficient ET mutants has as yet not been tested *in vivo*, there are discrepancies as to the adjuvanticity of catalytically inactive CyaA mutants, which have been proposed to result from a number of factors, including the genetic background of the mouse strain, the route of antigen delivery, the dose of CyaA mutant and the vaccination schedule [14],[15],[52],[53]. Genetically detoxified mutants of other cAMP elevating toxins such as CT or LT-I, or their individual B (binding) subunits, have been demonstrated to retain adjuvant activity [10], indicating that both cAMP production and toxin binding to specific receptors contribute to their adjuvanticity. This possibility has been ruled out for ET, as PA does not harbour any activity either on APC or T cells *in vitro* nor acts as an adjuvant *in vivo* [16]. On the other hand, at variance with other reports [15],[53], an enzymatically deficient CyaA mutant, highly purified to rule out a contamination by LPS, has been reported to display adjuvant properties comparable or even superior to wild-type CyaA [14],[52]. A potential implication of CD11b/CD18 in the adjuvanticity of CyaA appears unlikely, as this integrin suppresses cytokine production by dendritic cells [54],[55], and moreover does not account either for the immunodeviating activity of CyaA on APC *in vitro*, which is cAMP dependent [15],[18],[19], or for the conflicting results obtained by different groups *in vivo* [14],[15],[52],[53]. An integrated and detailed analysis of the structural and functional interaction of adenylate cyclase toxins with the different cellular components which together orchestrate the immune response is expected not only to clarify their mechanism of adjuvanticity but also to lead to the development of more specific and effective adjuvants.

## Materials and Methods

### Cells, antibodies, reagents, and toxins

Peripheral blood mononuclear cells were purified from buffy coats from anonymous healthy donors (collectively ∼30, available from authorised blood banks) by density gradient centrifugation on Ficoll-Paque (Amersham Biosciences, Buckinghamshire, UK), using a Beckman GS-6R tabletop centrifuge (Beckman Coulter SpA, Milan, Italy). Cells were washed 2× in phosphate buffered saline (PBS), resuspended in RPMI 1640 (Invitrogen Ltd, Paisley, UK) (buffered with sodium bicarbonate to pH 7.2) supplemented with 7.5% fetal calf serum (FCS) (Hyclone, Thermofischer Scientific Inc, SouthLogan, UT), plated in plastic flasks (Sarstedt AG, Numbrecht, Germany) and incubated overnight at 37°C in a humidified atmosphere with 5% CO_2_. Non-adherent cells, which consisted principally of peripheral blood lymphocytes (PBL) and of which >90% were T cells (CD3^+^), were centrifuged at 800×*g* for 5 min at room temperature in Beckman GS-6R tabletop centrifuge and resuspended in fresh RPMI 1640 supplemented with 7.5% FCS.

For cAMP measurement, T cells were purified from peripheral blood mononuclear cell suspensions using the StemSep Human T cell enrichment kit (Voden Medical Instruments SpA, Milan, Italy).

Phosphospecific antibodies recognizing the phosphorylated active forms of CD3ζ, ZAP-70 (Y493), Vav1 (Y160), Akt1 (T308/S473), Erk1/2 (T202/Y204), p38 (T180/Y182) and CREB (S133), as well as an antibody recognizing phosphorylated Y505 on Lck, were from Cell Signaling Technology (Beverly, MA), Santa Cruz Biotechnology (Santa Cruz, CA) and Biosource Europe SA (Nivelles, Belgium). An antibody against the phosphorylated PKA consensus phosphorylation site, R-X-X-pT-X-X/R-R-X-pS-X-X, was purchased from Cell Signaling Technology. Anti-CD3ζ, -Lck, ZAP-70, -Vav, -Erk2, -p38, -CREB and anti-actin antibodies were from Santa Cruz Biotechnology, Upstate Biotechnology (Dundee, UK) and Cell Signaling Technology. A mAb suitable for immunoprecipitation of tyrosine phosphorylated CD3ζ was kindly provided by M. Banyiash. Fluorochrome-labeled anti-CD3 mAb were obtained from Becton Dickinson Biosciences (Milan, Italy). Unlabeled secondary antibodies were purchased from Cappel (ICN Pharmaceuticals Inc, CA) and peroxidase labeled antibodies from Amersham Biosciences. IgG antibodies from OKT3 (anti-CD3; American Type Culture Collection, Manassas, VA) hybridoma supernatants were purified on Mabtrap (Amersham Biosciences, Inc) and titrated by flow cytometry.

CyaA and the enzymatically inactive variant CyaA-E5 (resulting from a Leu-Gln dipeptide insertion between D188 and I189 in the catalytic core of the enzyme) were expressed in *E. coli* and purified to near homogeneity by previously established procedures modified as described [56] in order to eliminate most of the contaminating endotoxin. The specific activity of CyaA, measured as described in Ladant *et al.* [26] was higher than 500 µmol cAMP/min.mg whereas CyaA-E5 had no detectable enzymatic activity. In both preparations the endotoxin content, determined using a LAL assay (QCL-1000 kit from Lonza), was below 0.5 EU/µg protein. PA, LF and EL1 were expressed in *E. coli* and purified as described [25],[45],[57]. H89, KT5720, IBMX and 8-CPT were purchased from Sigma-Aldrich (Milan, Italy) and Calbiochem (Merck Biosciences GmbH, Schwalboch, Germany).

### Cell activations and lysis, immunoblots

For immunoblot analyses cells were plated at 5×10^6^ cells/ml in plastic flasks in RPMI 1640 supplemented with 7.5% FCS and 2 mM CaCl_2_ (required for CyaA entry into the cells), added with CyaA/CyaA-E5, and incubated at 37°C in a humidified atmosphere with 5% CO_2_ for 2 h before activation. Alternatively, cells were plated as above, added with ET (ratio PA∶EF 1.6), and incubated at 37°C for 6 h before activation. Activations by TCR/CD3 cross-linking were performed by incubating PBL with saturating concentrations of anti-CD3 mAb (as assessed by flow cytometry) and 50 µg ml^−1^ secondary antibodies (goat anti-mouse immunoglobulin Ig) in RPMI 1640 for 1–5 min at 37°C as previously described [58]. None of the above mentioned treatments affected cell viability, as assessed by Trypan blue exclusion (data not shown). When required, cells were pretreated with the PKA inhibitors, H89 (20 µM) and KT5720 (56 nM), or with the PDE inhibitor, IBMX (0.5 mM), for 1 h before addition of ET or CyaA. Alternatively, cells were treated for 30 min with 8-CPT (100 µM).

Cells were recovered by centrifugation at 16,000×*g* for 30 sec at 4°C in an Eppendorf 5415R microcentrifuge (Eppendorf srl, Milan, Italy), washed 2× in PBS and lysed in 1% (v/v) Triton X-100 in 20 mM Tris-HCl pH 8, 150 mM NaCl (in the presence of 0.2 mg/ml Na orthovanadate, 1 µg/ml pepstatin, leupeptin, and aprotinin, and 10 mM phenyl methyl sulfonyl fluoride). To normalize for variations in protein content among samples, equal amounts of proteins from each sample (measured using a kit from Pierce, Rockford, IL) were resolved by 12% SDS-PAGE and transferred to 0.45-µm nitrocellulose filters Whatman GmbH, Dassel, Germany). Prestained molecular mass markers (Invitrogen) were included in each gel.

Immunoblots were carried out using primary antibodies and peroxidase-labeled secondary antibodies according to the manufacturers' instructions and a chemiluminescence detection kit (Pierce). Blots were scanned using a laser densitometer (Duoscan T2500 Agfa, Milan, Italy) and quantified using the ImageQuant 5.0 software (Molecular Dynamics, Sunnyvale, CA). Data were normalized to loading controls.

### T cell proliferation assays, cytokine, and cAMP measurements

For proliferation assays, cells (2×10^5^/sample) were plated in 96-well plates in RPMI 1640 supplemented with 7.5% FCS (and 2 mM CaCl_2_ for CyaA treatments and respective controls), added with CyaA/CyaA-E5 (0.07–45 nM) or ET/EL1 (0.01 pM–1.1 nM), and incubated at 37°C in a humidified atmosphere with 5% CO_2_ for 2 h (CyaA) or 6 h (ET) before activation. Cells were activated by CD3 cross-linking on secondary antibody-coated plates as described [58] and processed 16–48 h after activation. [^3^H]-thymidine (1 mCi) was added to each microtiter well (96-well plates) for the last 18 h of culture. After harvesting the cells with an automatic harvester (Micromate 196, Canberra Packard, Meriden, CT), proliferation was determined by measuring the [^3^H]thymidine (Amersham, Buckinghamshire, UK) incorporation in a β-counter (Matrix 9600, Canberra Packard, Meriden, CT).

To measure Th cell differentiation, enriched human CD4^+^ T cells were activated by immobilized anti-CD3 mAb (purified from OKT3 hybridoma supernatants on Mabtrap, Amersham Biosciences Europe) as described [59], in the absence or presence of recombinant hIL-12 (2 ng/ml, Sigma-Aldrich Milan, Italy) to promote Th1 differentiation, or recombinant hIL-4 (10 ng/ml, Sigma-Aldrich Milan, Italy) to promote Th2 differentiation. Recombinant hIL-2 (kindly provided by Eurocetus Milan, Italy) was added to the cultures on day 4 and 7. After 10 days, cells (1×10^6^) were washed, stimulated for 24 h or 48 h using anti-CD3 mAb and the levels of IL-4, IL-13, IFNγ and TNF-α were measured by ELISPOT as described [59].

Intracellular cAMP was quantitated by enzyme-linked immunoassay kit (Biotrak EIA, Amersham Biosciences) according to the manufacturers' instructions. For these experiments, cells (1×10^6^ plated in 96-well plates in 200 µl RPMI 1640/7.5% FCS) were treated with CyaA/CyaA-E5 or ET/EL1 as described above for 30 min to 24 h in a humidified atmosphere with 5% CO_2_. At the end of the treatment, cells were washed 2× in PBS and lysed in the lysis reagent included in the kit.

### RNA purification and real-time quantitative RT–PCR

Total RNA was extracted from Th cells, polarized as described above, using Tri Reagent (Ambion, Austin, TX). Reverse transcription-polymerase chain reaction (RT-PCR) was carried out on 400 ng total RNA using ImProm-II™ reverse transcriptase and and oligo-dT (Promega Italia srl, Milan, Italy) as first strand primer.

Real-time quantitative PCR was performed using SYBR Green I SensiMix™ dT Kit (Quantace, Watford, UK) according to the manufacturer's instructions, in an Opticon 2 Continuous Fluorescence Detection System (MJ Research, Bio-Rad Laboratories, Waltham, MA). All samples were run in duplicate on 96-well optical PCR plates (Roche Diagnostics, Milan, Italy). The specific primers used to amplify cDNA fragments corresponding to c-maf, GATA-3, T-bet and GAPDH were: 5′-TGGAGTCGGAGAAGAACCAG-3′ (*sense*), 5′-GCTTCCAAAATGTGGCGTAT-3′ (*antisense*) for c-Maf; 5′-GAAGGAAGGCATCCAGACCAG-3′ (*sense*), 5′-ACCCATGGCGGTGACCATGC-3′ (*antisense*) for GATA-3; 5′-TAATAACCCCTTTGCCAAAGG-3′ (*sense*); and 5′-TCCCCCAAGGAATTGACAGT-3′ (*anti-sense*) for T-bet and 5′-TGCACCACCAACTGCTTAGC-3′ (*sense*) and 5′-GGCATGGACTGTGGTCATGAG-3′ (*anti-sense*) for GAPDH. After an initial denaturation for 10 min at 95°C, denaturation at the subsequent 40 cycles was performed for 15 s at 95°C, followed by 15 s primer annealing at 60°C and a final extension at 72°C for 30 s. The ΔΔC_T_ method [60] was applied as a comparative quantification method. The specificity of the amplified fragment was demonstrated by the melting curve, where a single peak was observed for each sample amplified with c-maf, GATA-3, T-bet and GAPDH primers. c-maf, GATA-3 and T-bet mRNA levels were normalized to GAPDH, used as a housekeeping gene.

### Statistical analyses

Mean values, standard deviation values and Student's *t* test (unpaired) were calculated using the Microsoft Excel application. A level of *P*<0.05 was considered statistically significant.
